# Calcium Silicate-Based Biocompatible Light-Curable Dental Material for Dental Pulpal Complex

**DOI:** 10.3390/nano11030596

**Published:** 2021-02-27

**Authors:** Sung-Min Park, Woo-Rim Rhee, Kyu-Min Park, Yu-Jin Kim, Junyong Ahn, Jonathan C. Knowles, Jongbin Kim, Jisun Shin, Tae-Su Jang, Soo-Kyung Jun, Hae-Hyoung Lee, Jung-Hwan Lee

**Affiliations:** 1 Institute of Tissue Regeneration Engineering (ITREN), Dankook University, 119 Dandae-ro, Cheonan 31116, Chungcheongnam-do, Korea; ismpark1@naver.com (S.-M.P.); yujin911031@daum.net (Y.-J.K.); ajy3809@naver.com (J.A.); pedoshin@dankook.ac.kr (J.S.); 2 UCL Eastman-Korea Dental Medicine Innovation Centre, Dankook University, 119 Dandae-ro, Cheonan 31116, Chungcheongnam-do, Korea; j.knowles@ucl.ac.uk; 3 Department of Nanobiomedical Science & BK21 PLUS NBM Global Research Center for Regenerative Medicine, Dankook University, 119 Dandae-ro, Cheonan 31116, Chungcheongnam-do, Korea; 4 Department of Biomaterials Science, College of Dentistry, Dankook University, 119 Dandae-ro, Cheonan 31116, Chungcheongnam-do, Korea; woorim.rhee@gmail.com (W.-R.R.); pkmin0204@naver.com (K.-M.P.); 5 Division of Biomaterials and Tissue Engineering, University College London Eastman Dental Institute, London WC1X 8LT, UK; 6 Department of Pediatric Dentistry, College of Dentistry, Dankook University, 119 Dandae-ro, Cheonan 31116, Chungcheongnam-do, Korea; jbkim0222@dankook.ac.kr; 7 Department of Pre-medi, College of Medicine, Dankook University, Cheonan 31116, Chungcheongnam-do, Korea; jangts@dankook.ac.kr; 8 Department of Dental Hygiene, Hanseo University, 46. Hanseo 1-ro, Haemi-Myun, Seosan 31962, Chungcheognam-do, Korea

**Keywords:** calcium silicate, light-curable MTA, pulp regenerative dental materials, nanoparticle, odontogenic differentiation

## Abstract

Dental caries causes tooth defects and clinical treatment is essential. To prevent further damage and protect healthy teeth, appropriate dental material is a need. However, the biocompatibility of dental material is needed to secure the oral environment. For this purpose, biocompatible materials were investigated for incorporated with dental capping material. Among them, nanomaterials are applied to dental materials to enhance their chemical, mechanical, and biological properties. This research aimed to study the physicochemical and mechanical properties and biocompatibility of a recently introduced light-curable mineral trioxide aggregate (MTA)-like material without bisphenol A-glycidyl methacrylate (Bis-GMA). To overcome the compromised mechanical properties in the absence of Bis-GMA, silica nanoparticles were synthesized and blended with a dental polymer for the formation of a nano-network. This material was compared with a conventional light-curable MTA-like material that contains Bis-GMA. Investigation of the physiochemical properties followed ISO 4049. Hydroxyl and calcium ion release from the materials was measured over 21 days. The Vickers hardness test and three-point flexural strength test were used to assess the mechanical properties. Specimens were immersed in solutions that mimicked human body plasma for seven days, and surface characteristics were analyzed. Biological properties were assessed by cytotoxicity and biomineralization tests. There was no significant difference between the tested materials with respect to overall physicochemical properties and released calcium ions. The newly produced material released more calcium ions on the third day, but 14 days later, the other material containing Bis-GMA released higher levels of calcium ions. The microhardness was reduced in a low pH environment, and differences between the specimens were observed. The flexural strength of the newly developed material was significantly higher, and different surface morphologies were detected. The recently produced extract showed higher cell viability at an extract concentration of 100%, while mineralization was clear at the conventional concentration of 25%. No significant changes in the physical properties between Bis-GMA incorporate material and nanoparticle incorporate materials.

## 1. Introduction

Pulp-capping materials are necessary for protecting the defected pulp complex from thermal and chemical stimuli. Conventionally, mineral trioxide aggregate (MTA), which is a tricalcium silicate-based material that produces calcium hydroxide as a byproduct of hydration, is widely used as a pulp-capping material [1,2]. Despite the high biocompatibility and low solubility of MTA [3,4], the main drawbacks of MTA are that it takes a long time to set up and that it is difficult to handle when used in the clinical field. Therefore, light-curable calcium silicate-based pulp-capping materials have been investigated. Additionally, the light-curable MTA-like material releases Ca^2+^ ions and increases the alkalinity of the surroundings over time, which could induce the deposition of hard tissue by the formation of calcium hydroxide. In addition, the increased alkalinity creates an adverse environment for bacterial survival and proliferation, resulting in antibacterial effects [5]. Biological properties such as the release of hydroxyl ions, mineralization, and alkaline phosphatase activity are critical characteristics of successful pulp-capping materials [6]. Several studies have been conducted to identify whether MTA-like, light-curable pulp-capping materials can mineralize the peripheral environment compared to conventional MTA [5,7,8].

According to the information data sheet provided by the manufacturer, the light-curable MTA-like material contains 5–10% bisphenol A-glycidyl methacrylate (Bis-GMA) (Table 1). Bisphenol A, which is derived from Bis-GMA, is routinely used for the restoration of decayed, fractured, and poorly formed teeth. Despite the improved properties, such as strength, resistance, ease of application, translucency, and polishing ability, bisphenol A has been considered a harmful, estrogenic endocrine-disrupting material in several studies [9,10]. Bis-GMA, which is used in light-curable dental material, induces G2/M cell cycle arrest and apoptosis [11]. Additionally, Bis-GMA increases the number of progesterone receptors and reacts with receptors as a compatible substrate of estrogenic proteins [12,13]. To compensate for these issues, triethylene glycol- or dimethacrylate-containing Bis-GMA-free biopolymers have been investigated [14,15]. However, it is not suitable for clinical situations due to its weak physical strength [16].

Recently, various nanoparticles, such as carbon-based nanomaterials, hydroxyapatite, iron oxide, zirconia, silica, and titanium (TiO_2_), have been used in dental materials to enhance their physical, chemical, and biological properties [17]. Furthermore, a bioactive glass nanoparticle (BGN)-incorporated light-curable pulp-capping material with calcium hydroxide has been introduced and studied for its physical and biological properties. Studies have confirmed that the nanoparticles in the pulp-capping material enhanced the mechanical properties and biomineralization capacity [18,19]. Ceria nanoparticles (CNPs) have significantly elevated the odontoblastic differentiation of human dental pulp stem cells (hDPSCs) when included in commercially available MTA [20]. Graphene oxide nanosheets have been supplemented with Poly (methyl methacrylate) (PMMA) to give antimicrobial adhesive effects [21].

In this study, a new light-curable pulp-capping material incorporating 30–60 nm silica particles without Bis-GMA was studied. This material is similar to the previous light-curable MTA-like pulp-capping material with respect to the composition of tricalcium silicate. However, the absence of Bis-GMA is a distinct feature. This study showed the high biocompatibility of the recently produced light-curable material by biological tests, including a cell counting kit (CCK) assay, live and dead staining, alizarin red staining, and alkaline phosphatase activity. Chemical and physical properties were also investigated. The hypotheses of this study are that there are few significant chemical and physical differences between the conventional and new light-curable materials and that the new materials are superior to conventional materials.

## 2. Materials and Methods

### 2.1. Dental Materials

TheraCal LC (Bisco Inc., Schaumburg, IL, USA) is composed of type (iii) Portland cement, fumed silica, barium sulfate, barium zirconate, and resin containing Bis-GMA and Polyethylene glycol dimethacrylate (PEGDMA, Thermo Fisher Scientific, Waltham, IL, USA). The exact components of TheraCal LC were not informed by constructor policy.

Bright MTA capping (Genoss, Suwon, Korea) consists of calcium silicates (30–50%), polyethylene glycol dimethacrylate (10–30%), barium sulfate (15–30%), and silica nanoparticles (30–60 nm) (8–15%).

### 2.2. Physiochemical Properties

The valuation specimen’s depth of cure, solubility, and water absorption rate was followed by ISO 4049. TheraCal LC and bright MTA capping were filled in the mold (Ø = 4 mm, h = 1 mm) and light-cured for 20 s on the surface. The materials were light-cured using a 1700 mW cm^–2^ LED lamp (T-LED ELCA, Anthos, Imola, Italy) and removed from the mold. Each specimen was freeze-dried for seven days and measured the specimen’s weight (m_1_). Then, the specimens were immersed with deionized water at 37 °C for seven days and measured their weight once again (m_2_). After seven days passed, final dry was conducted to measure specimens’ weight (m_3_). These two different treated specimens were desiccated seven days more for measuring their changed weight (m_3_). The volume of the specimen was calculated in cubic millimeters (V). Solubility = (m_1_ − m_3_)/V. Water absorption = (m_2_ − m_3_)/V. Every measurement was repeated five times (n = 5).

### 2.3. Inorganic Amount and Size Distribution and Viscosity

The light-curable materials were polymerized in specific mold (Ø = 15 mm and h = 1 mm) and running thermogravimetric analysis (TGA) (TG 8121, Rigaku Corporation, Tokyo, Japan) to check decomposition rate by temperature changes (0–800 °C). After heating up to 800 °C, a laser particle size analyzer (LA-950, Horiba, Kyoto, Japan) was used to analyze the silica nanoparticle’s size. In addition, for optimization of dental materials of viscosity, viscometer was used and measured commercial dental material (TheraCal LC), silica nanoparticle incorporated bright MTA (bright MTA capping) and non-incorporated silica nanoparticle bright MTA (24.7 °C, n = 5) (VD1 CP-52, Brookfield, Toronto, ON, Canada).

### 2.4. Hydroxyl Ion (pH) and Calcium Ion (ppm) Release Test

The light-curable materials were cured on placed in the standardized mold (Ø = 15 mm and h = 1 mm). Cured specimens were placed on the bottom of a cylindrical container filled with 2.19 mL of deionized water and stored at 37 °C. After 1, 3, 7, 14, and 21 days, the storage water was removed and replaced as part of the test. The calcium ion release pattern was measured from media extracted from the dental material with inductively coupled plasma-atomic emission spectrometry (ICP–AES) (Optima 4300 DV, Perkin-Elmer, Waltham, MA, USA). Additionally, the, pH of the extraction media was detected with a digital pH meter (Orion 4 Star, Thermo Fisher Scientific). For accurate measurement, the blank control was detected first (7.6±0.1) and after performing calibration according to the manufacturer’s protocol. In each sample, three measurements (n = 3) were performed to confirm reproducibility. The average value (n = 3) ± SD was recorded.

### 2.5. Mechanical Properties

Bar-shaped specimens (2 mm × 2 mm × 25 mm) were immersed in solutions at pH 3, 4, 5.6, and 7.4 and stored at 37 °C. On day 7, the specimens were removed from the different pH solutions, after which they were washed and dried with air spray. The specimens were then polished using 1000-grit particle size sandpaper and positioned on a Vickers hardness machine (HM-211, Mitutoyo, Tokyo, Japan) to determine the hardness with 0.5 N (510 gf) for 20 s. The representative mean (n = 3) and standard deviation were calculated from three different locations on each specimen.

Light-curable materials were compacted into molds (2 mm × 2 mm × 25 mm) and light-cured for the manufacturer’s recommended time. Following light-curing, the specimens were immersed in 50 mL of distilled water for 24 h. For three-point flexural strength, a 100 N load cell (Instron 3344, Instron, Norwood, MA, USA) at a span length of 14 mm and loading rate of 1.0 mm/min was used. The flexural strength was determined from the relationship $FS=3Pl/2bd^{2}$, where l (mm) is the distance between the two supports, b (mm) is the width, and d (mm) is the depth of the specimen. The average value (n = 10) and standard deviation were calculated from 10 different tests conducted for each material.

The simulated body fluid (SBF) solution was used to mimic human body blood plasma ionic concentrations. SBF solution was obtained by dissolving NaCl (142.0 mM), KCl (5.0 mM), NaHCO_3_ (4.2 mM), CaCl_2_ (2.5 mM), MgCl_2_·6H_2_O (1.5 mM), K_2_HPO_4_·3H_2_O (1.0 mM), and Na_2_SO_4_ (0.5 mM) in distilled water buffered to pH 7.4 with Tris hydrochloride (Tris-HCl, Thermo Fisher Scientific). Two disc-shaped specimens (Ø = 15 mm and h = 1) were made for each material. One was immersed in SBF solution, and the other was immersed in DW. After one week at 37 °C, the surface morphology was observed by scanning electron microscopy (Sigma 300, ZEISS, Oberkochen, Germany). A magnification of ×10,000 was used to evaluate the different microstructural characteristics of the four different groups.

### 2.6. Cell Culture and Culture Conditions

Isolated non-carious third molars from dental patients were prepared for primary culture of human dental pulp stem cells (hDPSCs). The primary culture experimental protocol followed the Institutional Review Board (H-1407/009/004) and ISO 10993-5. The hDPSCs were cultured between the third and tenth passages for the definitive in vitro study. Alpha-minimum essential medium supplemented with 1% penicillin/streptomycin (Invitrogen, Carlsbad, CA, USA), 10% fetal bovine serum (Gibco, Waltham, MA, USA), 0.1 mM l-ascorbic acid (Sigma), and 2 mM GlutaMAX (Gibco) was used to maintain media to culture the hDPSCs (MM). The disk-formed dental materials (thickness = 2 mm and diameter = 6 mm) were immersed in cell culture media for making eluted media. To simulate the clinically adjustable environment, the eluates of all specimens were collected at 37 °C for 24 h in a shaking incubator (120 rpm).

### 2.7. Cell Viability Test

The cell viability test was performed according to ISO 10993-5. The 100%, 50%, 25%, 12.5%, 6.25%, and 0% concentrations extract media of two different dental material groups (bright MTA capping and TheraCal LC) were used to evaluate cell viability. The Cell Counting Kit-8 (CCK-8, Dojindo, Rockville, MD, USA) assay was performed based on the manufacturer’s protocol, and the results are presented as the optical density percentage. The analyses were performed in triplicate. The representative mean and standard deviation of each group are shown.

### 2.8. Cell Imaging

Confocal laser scanning microscopy (CLSM, LSM700, Carl Zeiss, Thornwood, NY, USA) was used to capture images of live and dead assays. The cell-permeable calcein-AM was stained for green fluorescence in the presence of intracellular esterase activity, which was retained within live cells. Dead cells lose their membrane integrity; thus, they were stained with red fluorescent ethidium homodimer-1.

### 2.9. Alizarin Red Staining (ARS) and Alkaline Phosphatase (ALP) Activity Assay

Alkaline phosphatase (ALP) activity assays and alizarin red staining (ARS) were performed to determine the in vitro odontoblastic differentiation of human dental pulp stem cells (hDPSCs). For the certain concentration of differentiation media (DM), 50 mg/mL ascorbic acid, 100 nM dexamethasone, and 10 mM β-glycerophosphate were added in eluted media. MM0 and DM0, as a negative and positive control, did not contain eluted media. In addition, The25, Br25 contained 25% of eluted media and 12.5 mg/mL ascorbic acid, 25 nM dexamethasone, and 2.5 mM β-glycerophosphate and 75% of DM to adjust the same composition of differentiation supplements. The The50 and Br50 made the same method based on the experiment media composition. At 14 days after differentiation, biomineralization measured by ALP activity was visualized by staining using a Sigmafast BGIP^®^/NBT tablet (Sigma) according to the manufacturer’s recommendations. On day 21 after differentiation, the cells were stained with a 2% ARS solution (pH 4.2) for 30 min. After staining and washing five times with DW, images were taken by light microscopy (Olympus IX71, Shinjuku, Tokyo, Japan). All analyses were performed in triplicate, and representative images are shown.

## 3. Results and Discussion

### 3.1. Physiochemical Properties of Nanoparticle-Incorporated, Light-Curable MTA

**Figure 1:**
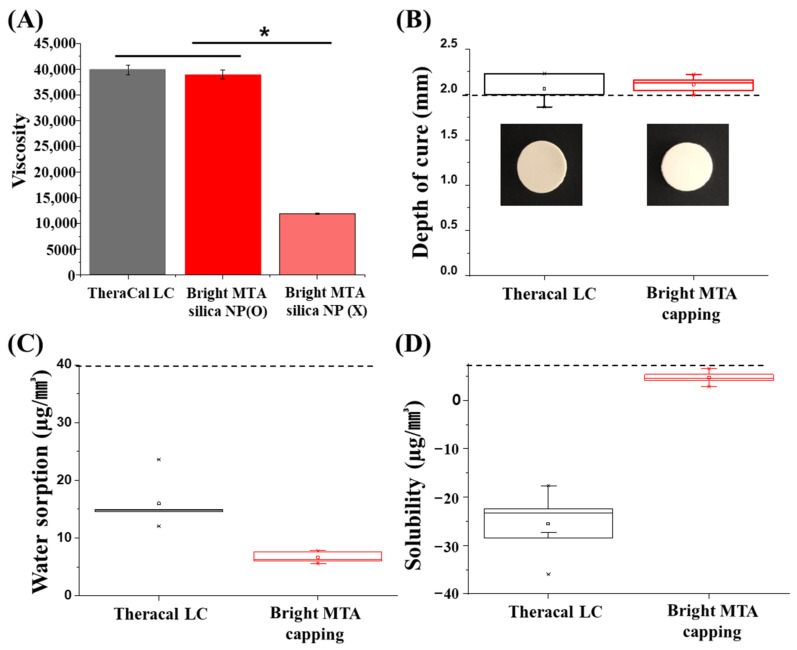
Physiochemical properties of TheraCal LC and bright MTA capping. (**A**) Viscosity comparison of TheraCal LC and bright MTA with silica nanoparticle (NP) or without (n = 5, at 24.7 °C *p* < 0.05). Asterisks (*) indicate statistical significance between specimen’s viscosity (*p* < 0.05). (**B**) Depth of cure (n = 5) of TheraCal and bright MTA capping was approximately 2.0 mm and 2.1 mm, respectively. According to ISO4049, opaque shade restorative materials are >2.0 mm. (**C**) Water absorption of TheraCal and bright MTA capping was 0.133 μg/mm^3^ and 0.066 μg/mm^3^ on average. According to ISO4049, at least four of the values obtained are ≤40 μg/mm^3^. (**D**) Water solubility (n = 5) of TheraCal and bright MTA capping was 0.025 μg/mm^3^, 0.004 μg/mm^3^ on average. According to ISO4049, at least four of the values obtained were ≤7.5 μg/mm^3^.

Bis-GMA is the finest material because of its high molecular weight and offering stiff properties in dental materials. However, leaving behind strength, poor biocompatibility has been issued. Here, to overcome these disadvantages, nano-silicate were investigated as a key component material that can provide similar physical properties instead of Bis-GMA. The conventional dental capping material called “TheraCal LC” and “bright MTA capping”, which incorporated nano-silicate were compared viscosity level and no significant differences were observed between them. However, when the fabrication was conducted without nano-silicate, the viscosity level showed too poor to use. It seems that nano-composition and nano-networking caused an increase in the viscosity of the bright MTA capping (n = 5, *p* < 0.05) (Figure 1A) [22,23]. When dental material is used in tooth defects, curing is necessary to cap the defect area [24]. The curing ability was examined based on ISO4049. Dental specimens were fabricated in disk-type formations for accurate experimental quantification. TheraCal LC was cured to an approximately 2.0 mm thickness, and bright MTA capping was cured to an approximately 2.1 mm thickness. Bright MTA capping cured slightly deeper than TheraCal LC (n = 5) (Figure 1B). Moreover, the specimen’s water absorption rate was measured by the standard of ISO4049. Due to the characteristics of dental materials, it is necessary to investigate changes in material properties caused by contact with moisture in the mouth [25]. From this point of view, the water absorption compared between two different dental materials was conducted. TheraCal LC absorbed more water than bright MTA capping. On average, TheraCal LC absorbed 0.133 μg/mm^3,^ and bright MTA capping absorbed 0.066 μg/mm^3^ (n = 5) (Figure 1C). Additionally, the consideration of solubility is necessary due to the durability of the dental material and the safety of the restored teeth [26]. From the ISO4049 standard, the solubility of the set materials should not exceed 40 μg/mm^3^, and no sample exceeded 40 μg/mm^3^. The high water absorption of TheraCal LC could be attributed to the incorporation of Bis-GMA. A previous study revealed that Bis-GMA leads to the formation of the most rigid network, which absorbs more water than the resin made by dimethacrylate-based materials [27]. However, ISO 4049 suggests that the solubility of the materials needs to be sufficiently less than 7.5 μg/mm^3^, and the values of TheraCal LC and bright MTA capping are both less than 7.5 μg/mm^3^ (n = 5) (Figure 1D). These results were considered to be due to the presence of Bis-GMA in the TheraCal LC. From the incorporation of Bis-GMA, a more rigid resin with a lower solubility value of the material was fabricated [27]. In fact, it is difficult to test detailed physical properties in consideration of each situation because there are various methods of use of dental materials in the clinical field. Moreover, there is a deficiency of international standards and test methods for resin-modified calcium silicate MTA-like cement.

**Figure 2:**
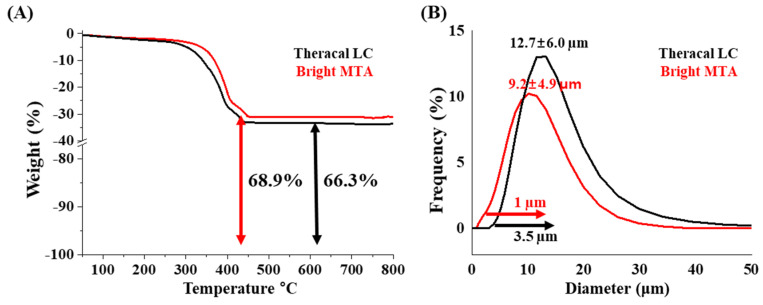
Inorganic amount and size distribution TheraCal LC and bright MTA. (**A**) Thermogravimetric analysis (TGA) results from 100 °C to 800 °C (n = 5) and (**B**) size distribution analysis after heat treatment at 800 °C (n = 5).

Thermogravimetric analysis (TGA) was used to identify the mass loss and characterization of their substances depending on an increase in temperature (up to 800 °C) [28]. The maximum weight loss occurred in the range of 300 °C to 400 °C in both materials. The total weight loss of bright MTA capping and TheraCal LC was 31.1% and 33.7%, respectively. This indicates that bright MTA capping contains more inorganic compounds (68.9%) than TheraCal LC (66.3%) (n = 5) (Figure 2A). The sizes of bright MTA capping and TheraCal LC, measured after heat treatment at 800 °C, was 9.2 ± 4.9 µm and 12.7 ± 6.0 µm, respectively (n = 5) (Figure 2B). The incorporated materials combustion was shown at high temperature (400–800 °C). [29,30,31]. However, nanosilicate-incorporated bright MTA capping can endure at high temperatures due to nanoscale network formation and inherent silicate characteristics. Further study is needed to understand the nanocomposite status.

### 3.2. Hydroxyl Ions (pH) and Calcium Ions (ppm) Released from Nanoparticle-Incorporated Light-Curable MTA

**Figure 3:**
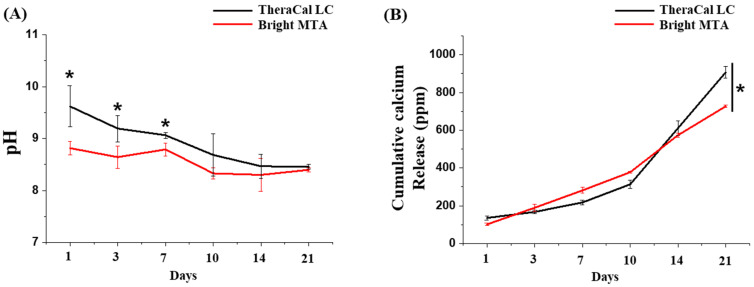
Ion release and pH changes from TheraCal LC and bright MTA capping over time. (**A**) The pH change was similar between TheraCal LC and bright MTA capping (n = 5). Asterisks (*) indicate statistical significance between bright MTA and TheraCal LC at the same pH (*p* < 0.05). The pH value fluctuated between pH 8 and 9 (*p* < 0.05, n = 5). (**B**) Calcium ions were similarly released for up to 10 days, with similar profile patterns between TheraCal LC and bright MTA capping, but after 10 days, TheraCal LC released more calcium ions than bright MTA capping (*p* < 0.05, n = 3). The ability of TheraCal LC and bright MTA capping to release calcium ions and alkalinize the surrounding fluids was correlated to the formation of calcium hydroxide Ca(OH)_2_.

After clinical treatment procedures, tissue inflammation causes changes in the pH of local defective sites [32]. The dental material should not change the pH due to the external environment. The dental material specimens were immersed in DW, and the change in pH was checked from 1 to 21 days. The TheraCal LC showed a higher pH than bright MTA from the beginning. TheraCal LC was already reported to give an alkaline pH after immersion in DW for three hours [33]. Due to its alkaline pH, it was susceptible to microbial infection and lead to greater inflammation [34,35]. This same phenomenon was observed in our study. The high pH value converged to pH 8 overtime (1–21 days). However, bright MTA capping showed a lower pH than TheraCal LC, which was steadily maintained. MTA capping showed a consistent pH change value so that the material properties might be maintained by the stimulus of external environments (n = 5) (Figure 3A). Additionally, to overcome dental hard tissue defects, calcium ion release from dental materials is important. From days 3 to 10, the release of calcium ions from the MTA capping appeared to be greater, but the release value was reversed afterward (n = 5) (Figure 3B). However, even high value of calcium ion release of TheraCal LC, the hard tissue restoration hindered due to the alkaline pH environment [33].

### 3.3. Mechanical Properties of Nanoparticle-Incorporated Light-Curable MTA

**Figure 4:**
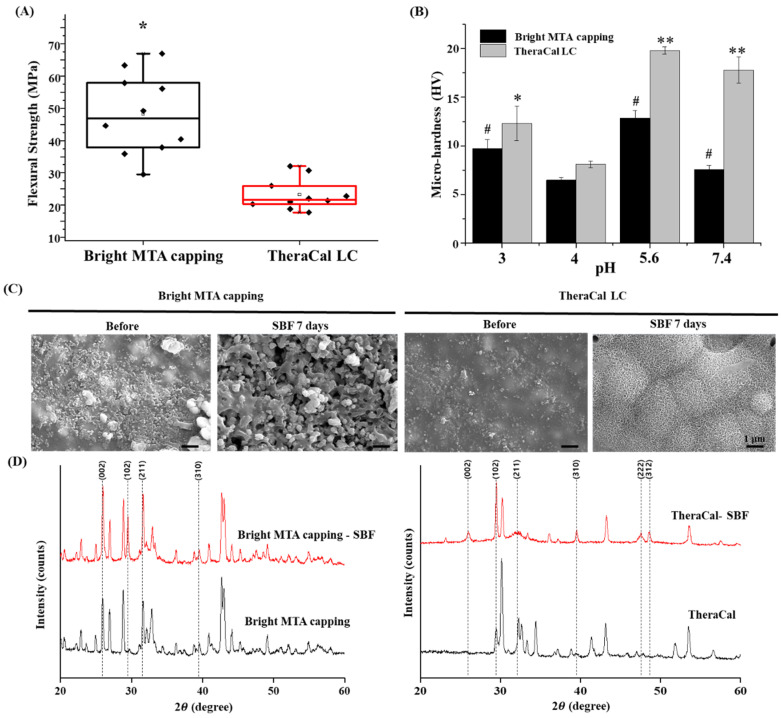
Mechano-physical properties of nanoparticle-incorporated, light-curable MTA. (**A**) Vickers hardness measured in five spots per specimen and averaged (n = 10). Asterisks (*) indicate statistical significance between bright MTA and TheraCal LC at the same pH (*p* < 0.05). Different letters indicate statistical significance between bright MTA and TheraCal LC at the same pH (*p* < 0.05). This test attempted to simulate the actual clinical environment during inflammation. (**B**) Three-point flexural strength (n = 10). There was a statistical difference between bright MTA and TheraCal LC. The (#) symbol and asterisks (*) indicate bright MTA capping and TheraCal LC’s statistical significance based on the lowest value of micro-hardness (* *p* < 0.05, ** *p* < 0.01) (pH = 4). (**C**) Surface morphology of bright MTA and TheraCal LC immersed in SBF solution for seven days, as visualized by scanning electron microscopy (×10,000) (scale bar = 1 μm). The SBF solution can mimic the main features of blood serum; thus, using the SBF solution can test bioactivity. (**D**) XRD analysis of specimens immersed in SBF for seven days.

With the use of dental material in clinical situations, the pH of dental environments can change for many reasons [34]. Additionally, the change of pH causes changes in material physical properties. Bright MTA capping showed a higher value of flexural strength than TheraCal LC under normal conditions (n = 10) (Figure 4A). In contrast, the microhardness value was higher in the TheraCal LC. The Bis-GMA content affects the microenvironment. Through low to high pH (3–7.4), TheraCal LC had a higher hardness than bright MTA capping, but a lower changing range of hardness was shown in bright MTA capping (n = 10) (*p* < 0.05) (Figure 4B). The dental material microhardness was significantly changed at low pH levels [35,36]. However, in clinical settings, the location of the dental material is kept at a low pH due to tissue inflammation. [34]. The materials that are stable under these various pH changes might be advantageous in clinical cases [35]. Additionally, the SBF assay was performed to mimic the clinical environment in which dental materials are used. The surface morphology embedded with SBF solution was visualized with SEM. Rough surfaces and a wide range of granule sizes appeared on bright MTA capping. However, TheraCal LC showed a clear hydroxyapatite microstructure, which was represented by a hair-shaped appearance (Figure 4C). The interaction between positively charged calcium ions from SBF and negatively charged silanol groups generates apatite nucleation formation [37]. It seems that the higher ability of crystal structure generation was performed in TheraCal LC, but it suggests that bright MTA capping can use of clinically [38]. Furthermore, the crystalline structure of hydroxyapatite was confirmed by XRD. The additional peak due to immersion in the SBF solution was shown in both dental material groups. However, the different peaks between the two dental materials were considered to be due to the incorporation of silica nanoparticles (Figure 4D). As a golden standard of light-curable capping material, TheraCal LC has a great possibility to affect dentin repair. In addition, the direct effect of nanoparticles was not shown in the odontogenesis experiment. However, it is not expected to provide a negative effect on the surrounding environment in the phase of regeneration. Calcium ion releasing pattern on early time point, and stable of pH change provide high calcium deposition and non-toxic circumstances. Moreover, the possibility of restoring the dental defect using bright MTA capping was shown, and improvements of bioactivities and adjust calcium ion release patterns are needed for better mechanical properties material.

### 3.4. Cell Viability and Odontoblastic Differentiation of Nanoparticle-Incorporated Light-Curable MTA

Tooth defects need to be filled with dental material to maintain their shape and protect from further damages. In addition, capping with dental material up to the pulp and dentin layer depends on the condition on the condition of the teeth. Thus, direct contact with materials and oral tissues needs to be considered. To mimic the environment in which dental materials are used, bright MTA and TheraCal LC were extracted for 24 h. The extracted media were diluted to different concentrations and co-cultured with hDPSCs (0–100%). High-concentration dental material extract media showed poor cell viability compared with low-concentration diluted media.

**Figure 5:**
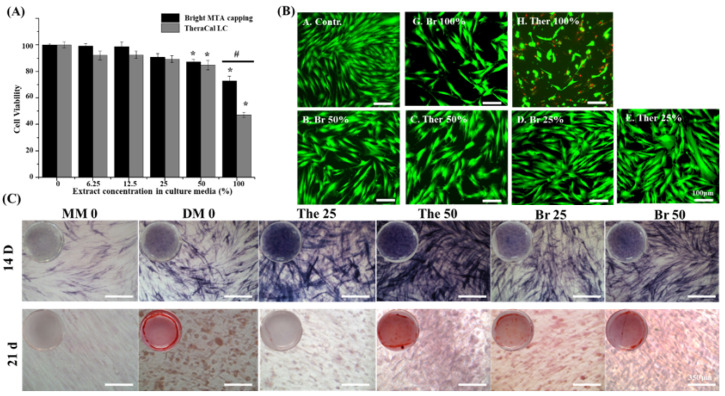
Cell viability and odontoblastic differentiation of nanoparticle-incorporated, light-curable MTA (**A**) Cell viability at various concentrations of extracts from TheraCal LC and bright MTA capping (n = 3, *p* < 0.05). Asterisks (*) indicate statistical significance between bright MTA and TheraCal LC’s cell viability based on the 0% of extract culture media. (#) the symbol indicates statistical significance between bright MTA and TheraCal LC’s cell viability at 100% extract culture media result. (**B**) Live and dead cells exposed to 25%, 50%, and 100% concentrations of extracts from bright MTA capping and TheraCal LC. Live (green) and dead (red) cells were observed by fluorescence microscopy (scale bar = 100 μm). Washing steps were performed before live and dead staining to ensure that only intact live cells remained on the plate (scale bar = 350 μm). (**C**) Odontoblastic differentiation under the use of Bright MTA and TheraCal LC was evaluated with alkaline phosphatase (ALP) activity (14 days) and calcium deposition (21 days) evaluated by alizarin red S (ARS) staining. ALP staining after 14 days revealed that 25% concentrations of extracts from both Bright MTA and TheraCal LC deposited a larger amount of phosphate than 50% concentrations. ARS staining after 21 days indicated a large amount of deposition in the 25% concentrations of extracts from both Bright MTA and TheraCal LC.

Incorporation of the Bis-GMA effect on cell viability resulted in half of the cells dying, as shown in the CCK-8 assay and live and dead assay [39,40,41]. However, the bright MTA capping extracted media did not show a significant cytotoxic effect on the hDPSCs (n = 3, *p* < 0.05) (Figure 5A,B). Moreover, the potential of hard tissue regeneration was proved by the odontoblast differentiation test from hDPSC [42]. To determine any adverse effects of elution media on bright MTA (Br) and TheraCal LC (The), odontoblast differentiation level was compared with maintaining media (MM) and differentiation media (DM). Odontoblast differentiated levels were observed with ALP staining and DM made more ALP positive cells than MM. However, two dental material eluted media showed more ALP staining than the normal media group (MM, DM). The calcium ions released from the dental materials were affected to differentiated cells [43]. The dental material eluted media was diluted in 25% (The25, Br25), 50% (The50, Br50) concentration, and the same number of odonto-differentiation supplements were added to check the calcium ion release effect. High concentrations of dental material elution media showed more ALP staining on day 14. Moreover, a similar phenomenon was displayed when checking the calcium deposition value using ARS staining at 21 days. Released calcium ions were affected the differentiated cells for up to 21 days. However, TheraCal LC eluted media showed higher effective-ness during differentiation of odontoblasts than bright MTA eluted media measured via the ALP staining. A similar differentiation effect was observed through ARS staining on day 21. Interestingly, the low concentration of bright MTA eluted media showed higher levels of differentiation at 21 days. The nano-silicate from bright MTA might influence cell differentiation and the calcium ion effect [44,45]. Both materials influenced the differentiation of hDPSC but showed better differentiation ability according to the calcium ion release pattern of TheraCal LC [46,47,48]. However, the higher cell viability of Bright MTA capping clearly suggests that there is no toxicity to the surrounding environment; hence, it is expected to help tissue regeneration in clinical treatment situations. For this purpose, a more detailed study is needed to investigate the mechanism of the relationship between eluted media and odontoblast differentiation.

## 4. Conclusions

In conclusion, the investigation of nano-silicate incorporated dental materials (Bright MTA capping) was conducted to enhance biocompatibility compare with Bis-GMA incorporated material (TheraCal LC). However, the nano-structure composition showed lower physical properties than Bis-GMA incorporated material. On the other hand, the stable pH change and initial calcium ion release pattern showed clinical applicability. In terms of dentin repair, the TheraCal LC’s alkalinization in the area of a dental defect will be an obstacle even with their great physical properties, although the other parameters indicative of regeneration of dentin tissue showed the superiority of TheraCal LC. However, creating a non-toxic environment at the site of the dental defect would be important for dentin repair. The bright MTA capping’s cell viability test and odontogenic marker level indicate a non-toxic effect on the surrounding environment. 

## Figures and Tables

**Table 1:** Summary of the difference between mineral trioxide aggregate (MTA)-like material (TheraCal LC) and bright MTA capping.

Product Name	Mixing Ratio	Method	Light Curing Time	Manufacturer	Composition (%)
Bright MTA capping	Single syringe (no-mix)	Light-curing	20 s	GENOSS (Korea)	Calcium silicate (30–50) Polyethylene glycol dimethacrylate (15–30) Barium sulfate (15–30) Silicate nanoparticles (8–15)
TheraCal LC	Single syringe (no-mix)	Light-curing	20 s	Bisco (USA)	Portland cement (30–50) Polyethylene glycol dimethacrylate (10-30) Barium zirconate powder (5–10) Bis-GMA (5–10)
